# Pre-operative Considerations in Adult Mucopolysaccharidosis Patients Planned for Cardiac Intervention

**DOI:** 10.3389/fcvm.2022.851016

**Published:** 2022-04-04

**Authors:** Benjamin Cross, Karolina M. Stepien, Chaitanya Gadepalli, Ahmed Kharabish, Peter Woolfson, Govind Tol, Petra Jenkins

**Affiliations:** ^1^Adult Congenital Heart Disease Department, Liverpool Heart and Chest Hospital, Liverpool, United Kingdom; ^2^Adult Inherited Metabolic Diseases Department, Salford Royal NHS Foundation Trust, Salford, United Kingdom; ^3^Ear Nose and Throat Department, Salford Royal NHS Foundation Trust, Salford, United Kingdom; ^4^Radiology Department, Liverpool Heart and Chest Hospital, Liverpool, United Kingdom; ^5^Radiology Department, Cairo University, Giza, Egypt; ^6^Cardiology Department, Salford Royal NHS Foundation Trust, Salford, United Kingdom; ^7^Anaesthetics Department, Salford Royal NHS Foundation Trust, Salford, United Kingdom

**Keywords:** heart disease, glycosaminoglycans, cardiac surgery, pre-operative assessment, adult MPS

## Abstract

Mucopolysaccharidoses (MPS) are rare lysosomal storage diseases characterized by multiorgan involvement and shortened longevity. Due to advances in therapies such as enzyme replacement therapy and haematopoietic stem cell therapy, life expectancy has increased posing newer challenges to patients and health professionals. One such challenge is cardiovascular manifestations of MPS, which can be life limiting and cause reduction in quality of life. Any cardiovascular intervention mandates comprehensive, multi-systemic work-up by specialist teams to optimize outcome. We highlight the importance of multidisciplinary evaluation of adult MPS patients requiring cardiovascular intervention. Clinical assessments and investigations are discussed, with a focus on the cardiac, anesthetic, airway, respiratory, radiological and psychosocial factors.

## Introduction

Mucopolysaccharidoses (MPS) are a heterogeneous group of disorders (type I, II, III, IV, VI, and VII) that result in the absence or deficiency of lysosomal enzymes, leading to an inappropriate storage of glycosaminoglycans (GAGs) and disruption of cell metabolism in various tissues of the body such as bones, heart valves, arteries, and nervous system (1).

Cardiovascular abnormalities affect up to 60-100% of MPS patients, especially those with MPS subtypes I, II, and VI (2). Cardiovascular pathology generally progresses earlier in life in those with more rapidly progressing subtypes of the disease (e.g., Hurler's syndrome). Cardiovascular disease is a progressive process across all subtypes with incidence and severity increasing over time (2). Despite severity, cardiac manifestations can be silent (2). Limited physical activity due to skeletal deformities and pain, and respiratory system involvement mask underlining cardiac insufficiency (3). In addition, individuals with cognitive impairment report fewer symptoms of cardiac disease.

Cardiovascular complications in adult MPS patients include cardiac valve infiltration commonly resulting in severe stenosis and/or regurgitation (2); conduction abnormalities (4–9), cardiomyopathy (10), pulmonary hypertension (3, 11, 12), coronary artery infiltration and other vascular involvement are rarer complications (2).

Enzyme replacement therapy (ERT) and haematopoietic stem cell therapy (HSCT) have been shown to improve the overall survival of MPS patients (13). The impact of HSCT and ERT on cardiovascular disease has been shown to reduce progression of left ventricular hypertrophy (2, 14) but neither influence valvular disease in reports in MPS I, II, and VI (15–19). However, HSCT has been reported to improve/stabilize valvular pathology in a study of MPS II in a Japanese cohort (19). With improved prognosis from ERT/HSCT therapy MPS patient survival has improved but cardiac valve disease progresses and has become more prevalent in adult MPS patients as their life expectancy improves.

Among new upcoming therapies, gene therapy has been shown to normalize the storage pathology in one animal study (20) and could have benefits in addressing cardiac valve disease in patients with MPS disorders.

With advances in new therapeutic developments for MPS disorders, there is an increasing need for better biomarkers of treatment effectiveness, which would help stratify the risk of cardiovascular disease progression. Apart from the GAG-related cardiac pathology, improved longevity of MPS patients imposes age-related cardiovascular complications, namely atherosclerosis.

Traditional biomarkers (GAGs), however, have not been shown to be useful in evaluating this risk of cardiovascular risk in adult MPS patients. It is also not clear whether monitoring of cardiovascular risk factors, including lipid profile, in MPS patients is useful in estimating their overall cardiovascular risk (21). However, progressively increasing B-Natriuretic Peptide (BNP) in asymptomatic valvular heart disease patients may point to advancing valve disease. BNP adds important incremental prognostic information that is useful for valve patient management and for optimal timing of surgery in particular. Its utility specifically in MPS is yet to be defined (10).

Successful cardiovascular interventions are becoming increasingly reported in MPS patients largely for aortic and mitral valve replacement (22, 23) however successful bypass, ventricular septal defect closure and corrective aortopathy procedures have been reported. As MPS patient survival continues to improve the need for cardiovascular interventions will increase, with some patients requiring multiple interventions in adulthood (22). Regular cardiac screening is required in these patients because symptoms may not occur or may be obscured. A summary of all the interventional/surgical procedures in MPS patients documented in the literature so far is shown in Table 1.

**Table 1:** Types of cardiac procedures in pediatric and adult MPS patients.

**Operative/interventional procedure performed**	**MPS type**	**Patient age in years/gender**	**References**
Mitral valve replacement	I-S	41 F	(24)
	I-H	16 F	(25)
	I-H/S	Unknown F	(26)
	II	33 M; 28 M; 25 M	(27–29)
	VI	25 F; 29 F	(30, 31)
Mitral valvuloplasty	III	6 F	(32)
Aortic valve replacement	I-S	62 M	(33, 34)
	II	10 M; 32 M	(34, 35)
	IV	31 F; 41 M; 60 F	(36–38)
	VI	43 M; 40 F	(39, 40)
Ross procedure	II	15 M	(41)
	IV	32 F	(42)
Aortic and mitral valve replacement	I	12 M	(43)
	I-S	23-35 M; 42 F; 52 M; 35 F; 49 F	(44–48)
	I-H	47 F, 39 F	(49)
	I-H/S	47 F; 20 M	(50, 51)
	II	18 M; 50 M	(52, 53)
	VI	21 F, 30 M, 34 F; 41 M; 42 M	(54–56)
Coronary artery bypass	I-S	56 M	(44)
Ventricular septal defect closure	III	15 M	(57)
Coarctectomy/repair	I-H	3 M	(58)
	VII	4 F	(59)
Resection of giant atrial appendage and mitral valve replacement	I-H/S	24 F	(60)
Aortic and mitral valve replacement with coronary bypass	VII	32 M	(61)
Konno aortoventriculoplasty with aortic root enlargement, patch plasty of a ventricular septal defect and RVOT and aortic valve replacement	I-H/S	Unknown F	(26)
Mitral, aortic and pulmonary valve replacement	II	62 M	(62)
TAVI	I-S	30 M	(63)
	II	50 M	(64)
Cardiac transplantation	II	23 M	(65)

*RVOT, right ventricle outflow tract; TAVI, transcatheter aortic valve implantation*.

In this review, we will focus on the cardiac and airway assessments required prior to cardiovascular intervention, multidisciplinary team working and psychological factors which should be considered prior to the procedure.

## Cardiovascular Evaluation

### Valvular Assessment

Valvular involvement is common in MPS, being present in almost all patients with MPS I, II, VI and to a lesser extent, other subtypes (2). The valvular disease occurs early, sometimes with rapid progression and high amounts of anatomical complexity due to involvement of both the valvular and sub valvular apparatus through GAG infiltration (10). Right sided pathology is much less common (2, 26) which is reflected in the available literature. Only one case report of pulmonary valve replacement as part of a three-valve replacement procedure has been reported (62). Subvalvular apparatus is also commonly involved often resulting in the tethering of the leaflets and their subsequently poor mobility which makes repair less likely to be successful and often favor replacement (26); however one case of MV repair is reported in the literature (32). The procedure most performed in the literature is aortic and mitral valve replacement in combination (see Table 1) and some surgical teams such as Rocha et al. (50) have suggested that aggressive preventive mitroaortic surgery should be performed even if the lesser affected valve functions with only mild-moderate disease to prevent re-sternotomy in such complex patients. Valvular intervention in MPS patients has many technical challenges including small annulae, small left ventricular outflow tracts and distorted anatomy. These technical aspects mandate highly specialist surgical techniques such as Ross-Konno Comando procedure (26, 60). It is imperative that a rigorous evaluation of valvular pathology is undertaken pre-operatively to plan optimum operative strategy.

Indications for cardiovascular intervention include (a) symptomatic valvular disease, which is uncommon and (b) asymptomatic individuals with severe valvular disease and with evident signs of cardiac compromise including systolic dysfunction, chamber dilatation, pulmonary hypertension and increasing frequency of arrhythmias (10). However, with no clear guidance on surgical “fitness” in MPS patients, they should be discussed on a case-by-case basis with a wide multidisciplinary team (MDT) including ENT airway experts, cardiothoracic anesthetists/intensivists, often neurosurgical teams for cervical spine issues, cardiologists/cardiothoracic surgeons and metabolic teams.

Echocardiography remains the mainstay of both screening and monitoring of valvular disease in MPS patients (10, 66). M-mode, 2-dimensional and Doppler echocardiography is the gold standard for the diagnosis of valvular involvement in MPS (10). It has been suggested that new technologies such as speckle tracking improve detection of subclinical left ventricular impairment including circumferential and radial strain as well as left ventricular twisting (67) albeit with minimal literature base. Valvular infiltration in MPS is common and has typical appearances on echocardiography (10). However, it should be kept in mind that spinal and chest wall deformities due to skeletal system involvement may limit this imaging modality (66) and adjustment for body surface area parameters in this cohort are essential. Transoesophageal echocardiography is not routinely employed in those with MPS due to poor tolerance and increased risks of the procedure under sedation and requirement for general anesthesia. Sedation or general anesthesia may be poorly tolerated because of the airway complications commonly seen in this patient population (2) making cross sectional imaging invaluable in valvular assessment in these patients (68, 69).

### Conduction Assessment

Conduction abnormalities have been reported in MPS patients (2, 8, 10). Of particular significance are the presence of atrioventricular blocks that have been reported in association with some MPS subtypes (II, III, VI, VII) which have been rarely reported to progress and associated with sudden cardiac death in some patients (6, 7, 70–72). Assessing for conduction abnormalities is essential in pre-operative assessment of these patients particularly when undergoing valvular surgery given atrio-ventricular (AV) block risks known in the general population are around 1.4% of all patients undergoing cardiac surgery (73) and up to around 1 in 12 (74) for surgical AV repair in particular. Transcatheter aortic valve implantation (TAVI) has been attempted and reported in a few case reports in MPS I and II (63, 64) and has the highest risk of post procedural AV block in the general population. Around 22% of patients undergoing TAVI have been reported to develop post-operative new-onset AV block requiring a permanent pacing device (75). As part of surgical assessment potential need for permanent pacing peri-operatively should be planned for and included in the consenting procedure for MPS patients.

### Coronary Assessment

In valvular surgery work up; coronary evaluation is mandatory for MPS patients (10); as it is for most valvular surgery patients (76). Successful valvular surgery with bypass and bypass grafting alone have been reported (44, 61), however remain infrequent in the literature likely largely due to absence of typical angina symptoms in this cohort (61). Evaluation for the presence of coronary arteriopathy however, in the MPS disorders may be problematic due to the diffuse nature of coronary involvement which differs substantially from typical atherosclerotic coronary disease (2). To our knowledge neither optical coherence tomography or intravascular ultrasound have been used in MPS cohorts to evaluate coronary disease however this has potential future clinical utility to aid assessment. CT coronary angiography is a very useful imaging modality pre-operatively in these patients.

### Aortic Assessment

Aortopathy is an emerging issue in adult MPS with the exact pathogenesis being unclear (77), but accumulation of GAGs have been implicated in increased vascular stiffness (78), aortic narrowing (2) and aortic root dilatation (77, 79). In a cohort of 34 MPS I-VII patients 35.3% developed aortic dilation, with the highest prevalence in MPS IVA (62.5%) (79) and 66% (80). A further cohort study of 69 patients demonstrated a prevalence of aortic root dilatation in 39.1% with the highest prevalence in MPS I-H and little effect of ERT on aortopathy (81). There are no specific indications for surgery for aortopathy in MPS (10) therefore usual thresholds and guidelines for aortic intervention should be followed (82). Due to complexities of this patient cohort discussion for intervention should be performed on a case-by-case basis (10). There are two reports of coartectomy/repair in the literature both of which were performed in early childhood for which the indication was to reduce secondary systemic hypertension to reduce risk of coronary artery disease and pressure on already vulnerable valves (58, 59). Medical management is emerging from lab mouse models where inhibition to renin-angiotensin system may help prevent aortic perturbation (83).

### Cardiomyopathies and Pulmonary Hypertension Assessments

#### Pulmonary Hypertension

The frequency and mechanisms of pulmonary hypertension (primary vs. secondary) are poorly classified in MPS patients. However, it does appear to be of significant burden to the MPS population; in one pediatric echocardiography study 10/28 patients were found to have pulmonary hypertension (11), supported by an adult cohort demonstrating pulmonary hypertension in around one in five patients with MPS (84). Implicated factors in the pathogenesis include left cardiac valve lesions (11), deposits of GAGs in pulmonary vascular bed (3, 11, 12), thoracic deformities, frequent pneumonias and obstructive apnoea (11, 85). Assessment of pulmonary hypertension is essential when planning cardiac interventions regardless of mechanism and in particular those that will require a general anesthetic due to the ventilatory and induction difficulties encountered with pulmonary hypertension (86). While there is little data in MPS literature around this; patients being investigated for valvular or coronary intervention will certainly have increased procedural risk if there is concomitant pulmonary hypertension.

#### Cardiomyopathy

Cardiomyopathy in MPS is an essential component of assessment as the etiology underlying may prompt interventions outlined above. Progressive, hypokinetic cardiomyopathy should raise the suspicion of an underlying ischemic disease (10). To the best of our knowledge, cardiac stenting has never been attempted in MPS cohorts; likely secondary to the diffuse nature of endovascular involvement from GAG deposition (2, 87) as well as technical challenges such as small caliber coronary arteries requiring pediatric catheters for access. However, coronary artery bypass graft revascularisation may be an option. Cardiomyopathy secondary to valvular disease generally follows a different and less severe clinical course with clear progressive valvular involvement which should prompt the referral for surgical assessment (10).

Primary cardiomyopathies appear to be less common in MPS patients than secondary (10) and likely are resultant directly from accumulation of GAGs within myocytes (2, 87). Ventricular hypertrophy is the most common primary cardiomyopathy seen in MPS patients (11, 45), in particular I, II, and VI subtypes (88, 89) with up to 50% of these groups demonstrating increased left ventricular mass (90). Electrocardiogram analysis for ventricular hypertrophy in this cohort is unlikely to be diagnostic as GAGs infiltration is non-conducting (11, 91), therefore reliance on imaging modalities such as echocardiography is essential. Reports of ventricular dilatation are less common (11) ranging from around 7-21% (11, 92). Dilated cardiomyopathy with early onset is considered the hallmark of aggressive MPS disease (87) and generally requires hospital admission for optimisation of heart failure with medical therapy (10). It is important to distinguish true dilated cardiomyopathy from that related to valvular dysfunction (91) which is likely seen in the higher prevalence paper by Gross et al. (92). Nevertheless, there is one report of true isolated dilated cardiomyopathy in MPS I (93). Insurance of assessment and treatment for reversible causes of acute cardiomyopathy such as myocarditis is an essential part of hospital management. Overall, there is growing evidence that ERT and HSCT therapies can cause stabilization and even regression of primary cardiomyopathies in MPS patients (15, 88, 90, 91) and therefore cardiac interventions for primary cardiomyopathies are less likely to be required compared with medical therapy.

Ultimate interventional management of both primary pulmonary hypertension and primary cardiomyopathies (dilated and hypertrophic) would include device therapy and transplant. To our knowledge biventricular pacing has never been attempted in MPS patient cohorts with heart failure. There has been an MPS case in the literature for heart transplantation which unfortunately was unsuccessful (65). Discussion around this case commented that the underlying cardiomyopathy was likely to have been secondary to valvular disease, underlining the importance of referral for valvular replacement. In general, unfortunately, most MPS patients are unlikely to be listed, have successful matching and subsequent transplant surgery for a multitude of reasons. Barriers to cardiac transplantation may include; mismatches in chest dimensions with cardiac donors, requirements for blood transfusions in prior management causing HLA sensitivity, complex chest wall anatomy, previous sternotomy, airway and C spine complexities and immunosuppression issues post operatively including recurrent respiratory infections and permanent intravenous access lines for enzyme replacement delivery increasing endocarditis risks.

### Cardiac Imaging

With the absence of cardiac symptoms for most adult MPS patients there is a large reliance on imaging modalities in the cardiologist's assessment of this patient cohort which comes with its own set of challenges.

The positions of Magnetic Resonance Imaging (MRI) and Computer tomography (CT) in MPS patient imaging guidelines are still unclear (Supplementary Table 2). Echocardiography remains the key diagnostic modality, while MRI and CT are excellent supplementary tools providing additional information.

#### MRI

**Figure 1:**
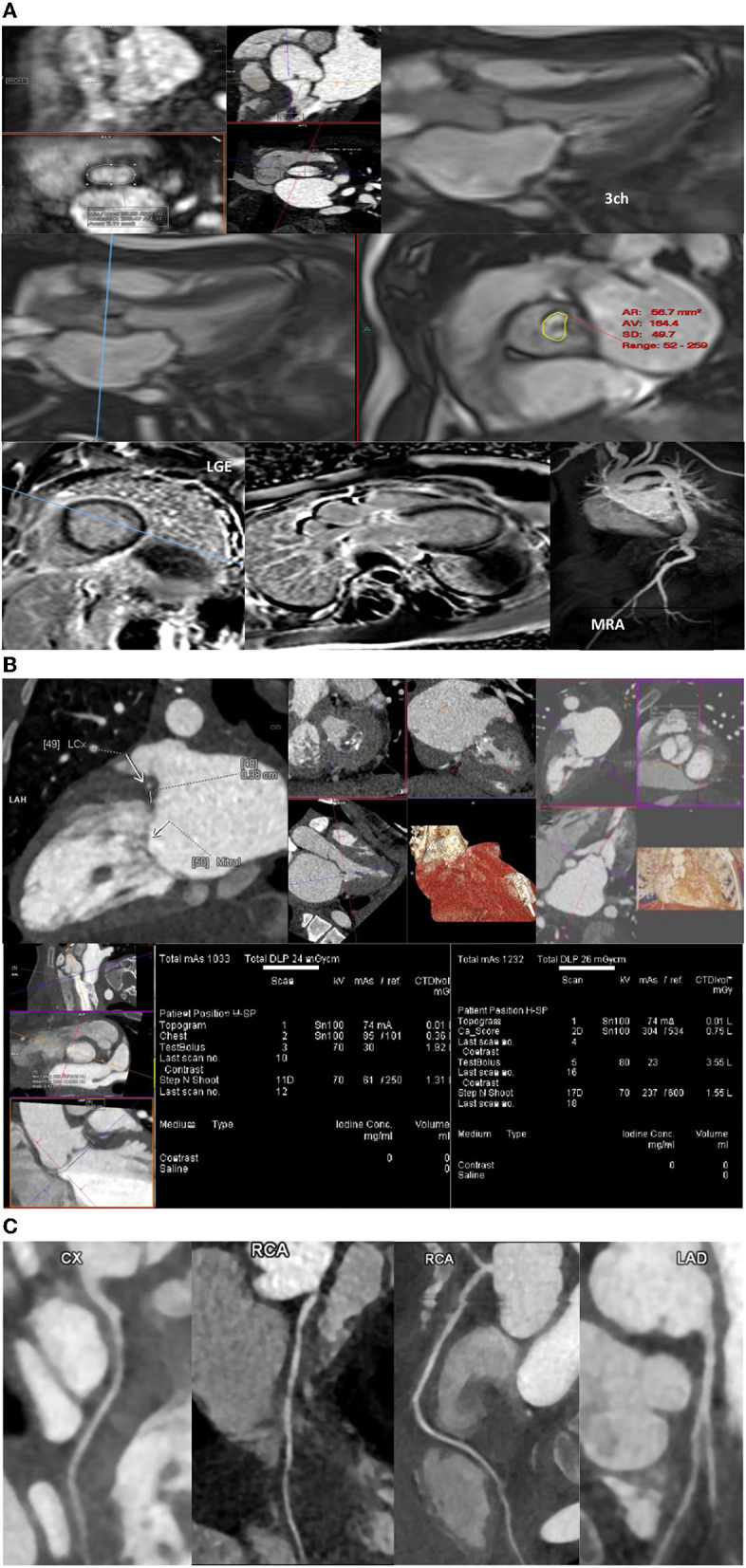
**(A–C)** Magnetic resonance imaging, computed tomography, and coronary magnetic resonance imaging in MPS patients.

Valvular involvement is frequently encountered in many patients with MPS (2) with left side valves more affected than right side heart valves, with the mitral valve being the most commonly affected (89, 94). MRI is superior to echocardiography in assessing cardiac volumes and flows in general and assessing valvular disease in particular (68). Therefore, MRI may be indicated for accurate baseline assessment of valvular lesion regurgitation fraction (RF%), accurately assessing cardiac volumes and functions preoperatively. Adjusted views may also help accurately assess annular sizes (Figures 1A–C). Valvular stenosis is not uncommon in patients with MPS. MRI may provide additional information necessary for planning an intervention (95). Cine views could assess valve morphology (bicuspid vs. tricuspid), leaflet thickening, degree of stenosis and valve area measurements (Figures 1A–C). Multilevel through- and/or in-plane flows can assess transvalvular maximum velocity. MRI can provide accurate assessment of indirect consequences of valvular stenosis, such as assessing hypertrophy through measuring myocardial muscle mass or left atrial volume. This will help with accurate planning and timing of intervention or surgery. Tissue characterization is among the strengths of MRI; it can provide insights to cardiac involvement in MPS. Gadolinium studies may reveal patterns of fibrosis that could be relevant preoperatively or assist in risk assessment of developing arrythmias. The relatively recent MRI-techniques such as T1 maps may indicate expansion of extracellular volume and deposition of abnormal material within the myocardial spaces.

Aorta and great vessels might be dilated or narrowed in patients with MPS (96). MR-angiography (MRA) can assess the aorta and femoral artery access suitability (Figures 1A–C) and will provide aortic diameters without radiation exposure (with or without contrast using respiratory navigated 3D sequences). The flow studies and cine sequences may detect and assess segments of stenosis or a coarctation if present.

#### CT

Various types of coronary artery disease (CAD) may occur in the different subtypes of MPS syndrome. Generally, CT is not routinely used for coronary evaluation in children. This is because of the unclear guidelines regarding the best method for evaluation of CAD in MPS and radiation exposure. Moreover, evaluation of CAD in MPS disorders is challenging because of the diffuse pathology. CT coronary angiography can be invaluable in pre-operative assessment, but interpretation must take into account difficulties with assessment because of fast heart rate blurring, or difficult interpretation/underestimation of disease due to diffuse coronary circumferential involvement (69).

## Airway, Anesthesia and Ventilation

All adult MPS patients being prepared for any cardiac intervention should be offered a detailed airway assessment. The common methods employed to assess airways and ventilation are detailed history, clinical examination, nasendoscopy, cross-sectional imaging, pulmonary function tests. In addition, 3-dimensional reconstruction, virtual endoscopy as additional tools can provide more information.

The assessments should be performed by obtaining a focussed history; probing any problems with airway, voice, swallowing, sleep apnoea, previous anesthetics, medications, allergies, previous medical conditions, neck mobility and mouth opening. The clinical examination should include mouth opening, dentition, modified mallampati grade (97), tongue bulkiness, thyromental distance (98), neck palpation, spine mobility and nasendoscopy. Nasendoscopy is an out-patient procedure where a flexible telescope is passed *via* nasal cavity into the pharynx enabling direct visualization of the oropharynx, larynx and part of hypopharynx. This examination has not only helped us to assess the airway but also help us plan awake fiberoptic nasal intubation in those patients where the mouth opening is limited. In the pharynx, the nasendoscopy allows assessment of the height of the larynx by estimating the distance between the epiglottis and tip of epiglottis; the bulkiness of the soft tissues of the supraglottis, dynamic collapse during inspiration and vocal cord mobility.

For ease of assessment, the airways can be divided into upper (oral cavity to level of glottis), central (sub-glottis, trachea) and lower airways (the bronchi, bronchioles, alveoli). The upper and central airways are evaluated by clinical examination, nasendoscopy, cross-section imaging; the lower airways were evaluated by pulmonary function tests such as FVC% and FEV% (Supplementary Table 3).

The various upper, central and lower airway findings can be used to assess 15 parameters to calculate the Salford Mucopolysaccharidosis Airway Score (SMAS) (99) to quantify the airway severity. SMAS questionnaire (Supplementary Table 4). This score enables to holistically assess airway and ventilation. A SMAS score of more than 25 can be considered a difficult airway and ventilation. Previous cardiac surgery can lead to vocal cord palsy (100), hence pre-operative nasendoscopy is important. Adult MPS I patients appear to have milder airway abnormalities to MPS II, MPS IV, MPS VI, and MPS VII (2, 99). MPS II have difficult upper, central and lower airways such as high larynx, bulky supraglottis, obstructive sleep apnoea, tracheomalacia, and low FEV1% and FVC% in addition to cervical spine problems. MPS IV, VI, and VII in addition have tortuous airways (2) (Supplementary Figures 2–5).

All adult MPS often manifest some form of airway abnormality (101, 102). Restricted mouth opening, prominent teeth, poor cervical spine mobility or unstable spine will make oral intubation difficult if not impossible. Airway complications are commonly seen in MPS I, II, IV, and VI and considerably contribute to morbidity and premature mortality (101, 102); this in the background of a cardiac disease makes clinical care complex. When treating adult MPS patients for cardiac disease, airway disorders have to be carefully considered.

### MPS and the Airway

High larynx, anterior larynx makes access to larynx very difficult. Metanalysis of 35 studies by Shiga et al. involving 50,760 patients revealed the incidence of difficult intubation is about 5.8 % in normal patients, 3.1% for obstetric patients and 14.8% in obese patients (103). This may be higher in MPS, it is important that this is kept in mind. Based on laryngoscopy views Cormack (104) graded the airway into three grades; grade 1 being full view of the glottis, grade 2—partial view of the glottis, grade 3—only epiglottis is visible, grade 4—neither epiglottis nor glottis are visible. In adult MPS cohort, laryngoscopy views 3 or 4 can be seen by applying far lateral approach from the oropharynx using a hokpins telescope or awake fiberoptic nasal intubation. A video laryngoscope in all adult MPS patients having general anesthetic is useful. Bonfils Retromolar Intubation Fiberscope® produced by Karl Storz–Endoskope, Germany is a very useful airway adjunct (105). Bulky airways such as large epiglottis and bulky supraglottis makes use of supraglottic airway devices such as laryngeal mask airways less useful. However, supraglottic airway with Trans nasal Humidified Rapid-Insufflation and Ventilatory exchange (THRIVE) (106) has proven to be useful in induction and recovery from anesthesia as were small endotracheal tubes such as a micro laryngeal tubes, micro cuffed tubes, un-cuffed tubes in tracheal intubation. Vocal cord mobility is very important in maintaining patent airway. Vocal cord palsy is a known complication following thoracic surgery (100, 107, 108), various mechanisms have been proposed (109). The resultant injury leads to reduction in quality of life due to dysphonia. This can also pose problems with airway post cardiac surgery. Hence immediate post cardio-thoracic surgery changes in voice and swallowing should be investigated for vocal cord paralysis.

### Associated Factors

Skin in MPS patients can be thick, making peripheral venous and arterial access difficult. Organomegaly from liver, spleen and chest wall deformity can splint the diaphragm limiting intra thoracic expansion of the lungs. Slightly upright position reduces the pressure over the diaphragm and improves ventilation. Similarly, lying down flat or some supine positions may not be possible due to spine, hip deformities.

Some patients also require ventriculoperitoneal (VP) shunts which increases cardiac surgery risks as entering the pleural space can cause VP shunt complications such as infection (26). Indwelling lines for ERT also increase the risk for endocarditis and surgical repair/replacement failure (39).

#### Anesthetic Plan

Induction should be performed in operating room environments under close surveillance of an otolaryngologist, experienced anesthetic and scrub team (110). Access to video laryngoscope, Hopkins telescope, various sizes of endotracheal tubes, supraglottic airway devices such as laryngeal mask airway, THRIVE, front of neck access equipment (39). The anesthetic plan includes several steps as follows:

*Step 1*

Intravenous access.

Bispectral index monitoring (BIS) (111).

Electrocardiographic monitoring.

Pre-oxygenation.

THRIVE (106).

Skin marking of cricothyroid membrane.

Awake fiber optic nasal intubation in co-operative patients following local anesthetic or intra venous induction in learning difficulties.

*Step 2*

Total intravenous anesthesia (TIVA), consider inserting nasopharyngeal airway.

Plan A: Video laryngoscopy, push tongue away to left.

– Access oropharynx, larynx via corner of mouth; insert a pediatric bougie and rail road a small endo tracheal tube.
– Access oropharynx, larynx via corner of mouth; insert a Hopkins telescope rail road a small endotracheal tube.

Failure in plan A → Insert a guedel oropharyngeal airway and nasopharyngeal airway → face mask ventilation, consider reverting to plan A or go to plan B.

Plan B: Maintain ventilation by supraglottic airway device such as small re-inforced laryngeal mask airway. Once stable, consider plan A.

Failure in plan B → Go to plan C.

Plan C: Insert a guedel oropharyngeal airway, nasopharyngeal airway and face mask ventilation.

Once stable consider plan A or B.

Failure in plan C → Consider plan D.

Plan D: front of neck access; incise the cricothyroid membrane, pass a pediatric bougie and pass an appropriately sized endo tracheal tube.

Once stable, consider plan A.

If not stable to proceed with surgery – abandon surgery and recover.

*Step 3: Recovery*

Following surgery, patient may be recovered in the operating theater or in the intensive care unit as appropriate.

– Leak test, cuff deflated and leak around the endotracheal tube assures no airway oedema.
– Recover patient with THRIVE or laryngeal mask airway or room air with nebulised adrenaline.
– Head up or sitting up may help to reduce the splinting of diaphragm.
– Expect increased secretions from throat and upper airways, managed by saline nebulisers and suction.

## Psychosocial Factors

MPS patients manifest with neurocognitive impairment (112), poor vision (113), hearing problems (114) which can become a communication barrier, leading to social isolation. In clinical practice it impacts the patient-health professional relationship.

Cardiac disease emerges silently and contributes significantly to early mortality, often suddenly. Patients and their families are aware that if untreated, cardiopulmonary complications are the main cause of mortality in MPS disorders, which results in their anxiety and depression.

Significant co-morbidities and the advanced cardiac disease are the factors for the cardiac surgery to be carefully considered. Cardiorespiratory complications, including airway difficulties, account for 63% of mortality among MPS patients peri-operatively, including 4.2% 30-day mortality for MPS I only (115, 116).

The health-related quality of life is low in MPS patients (117), hence any intervention should be carefully considered weighing the risks and benefits. The multidisciplinary approach proves to be helpful in making complex decisions. In particular, a best interest meeting is essential when clinical decisions are made on behalf of adults with limited capacity. Importantly, the long-term cardiology surveillance is required to monitor the disease progression in the context of the disease modifying therapy.

## Conclusions

Adult MPS disorders are often complicated by cardiovascular disease, requiring cardiovascular intervention. The perioperative assessment is complex and involves several specialists, including those with experience in managing pediatric and adult MPS disorders. Patients and their families must be actively involved in every stage of the peri-operative MDT and their views should be always considered. With the era of upcoming therapeutic advances and development of new cardiovascular interventional procedures and technologies, there are new opportunities for cardiovascular research with the aim to improve adult MPS patients' prognosis and quality of life.

## Author Contributions

BC, KS, PJ, and CG were involved in designing the concept of the study and oversight. BC, CG, AK, and KS drafted the manuscript. BC, KS, CG, GT, AK, PJ, and PW contributed to the acquisition and interpretation of all data. All authors contributed to the overall analysis of the results and in writing and reviewing the manuscript and have read and approved the final manuscript.

## Funding

The article processing fee was supported by BioMarin Pharmaceutical Inc. The funder was not involved in the study design, collection, analysis, interpretation of data, the writing of this article or the decision to submit it for publication.

## Conflict of Interest

The authors declare that the research was conducted in the absence of any commercial or financial relationships that could be construed as a potential conflict of interest.

## Publisher's Note

All claims expressed in this article are solely those of the authors and do not necessarily represent those of their affiliated organizations, or those of the publisher, the editors and the reviewers. Any product that may be evaluated in this article, or claim that may be made by its manufacturer, is not guaranteed or endorsed by the publisher.

## Abbreviations

AV: atrio-ventricular
BNP: B-Natriuretic Peptide
CAD: coronary artery disease
CT: computed tomography
MDT: multidisciplinary team
MPS: mucopolysaccharidosis
MRA: magnetic resonance angiography
MRI: magnetic resonance imaging
TAVI: transcatheter aortic valve implantation.
