# Supplementary material for: Pre-operative Considerations in Adult Mucopolysaccharidosis Patients Planned for Cardiac Intervention
Source: Front Cardiovasc Med. 2022 Apr 4;9:851016. doi: 10.3389/fcvm.2022.851016 (PMC9013828; doi:10.3389/fcvm.2022.851016)
Supplement: Supplementary file 1 [file Table_1.docx]

| Table 1 Supplementary. Clinical indications for MRI, CT in adult MPS |
| --- |
| Clinical indications for MRI include:   1. A baseline comprehensive segmental description of cardiac anomalies that might coexist. 2. MRA aorta to identify aortopathy and provide reliable aortic dimensions that could be used during follow up. 3. A baseline comprehensive preoperative valvular and functional study of;    1. Cardiac functions and volumes.    2. Type and severity of existing valvular lesions.    3. Non-invasive tissue characterization and early detection and quantification of scarring if present. 4. MRI could be performed post operatively to determine myocardial remodelling (positive / negative) and assess outcome.   Clinical indications for CT in MPS include:   1. CT coronary may be indicated when strongly suspecting CAD (52) If HR still high or irregular; acquisition in a systolic phase (isovolumetric phase around 40%) can be performed with good quality (figure 3). Padding and pulsing techniques could be used to acquire different cardiac phases, but they might lead to increased radiation. 2. Cardiac CT preoperative planning:    1. Accurate annular sizing.    2. Coronary anatomical relations to valves in patients with planned valve replacement (figure 2).    3. Coronary anatomy (figure 2).    4. Degree of valve /coronary calcification.    5. Aortic dimensions.    6. Functional CT to assess cardiac functions in patients with pacemaker and poor echo windows.   General recommendations for MRI and CT protocol in patients with MPS:   1. A comprehensive baseline valvular, functional, and anatomical MRI study, to evaluate anatomy, heart functions, aorta (without contrast) and valves. 2. Contrast MRI late gadolinium study when appropriate, for instance in presence of left ventricular dysfunction or recent arrythmias. 3. CT coronary (with adjusted parameters) with a strong suspicion of CAD or progressive cardiac impairment. Adenosine stress MRI may represent a potential alternative to testing CAD in MPS. 4. If a surgery / intervention decision by the multi-disciplinary team is planned: a combination of a cardiac CT and aorto-femoral MRA with contrast would be advisable. Cardiac CT is advised for accurate annular sizing, coronary anatomical relations, etc. while MRA assesses femoral access without excess radiation exposure.   Safety considerations:   - 1. Severe aortic stenosis may exist in some patients with MPS, where administration of beta blocker is sometimes contraindicated. Communication with the referrer is highly advisable to discuss the use of BB or alternative medications to control heart rate (HR) e.g. Ivabradine.   2. Amount of contrast must be calculated carefully according to body weight (max. 0.5-0.2ml contrast /kg). Knowing the severity of valvular stenosis beforehand is paramount to avoid fluid volume overload.   3. In addition, avoidance of multiple tests and studies on same date is advisable to avoid patient exhaustion.   4. Many patients with MPS have vertebral abnormalities and cannot tolerate lengthy scan times, and the occasional need for sedation. Targeted scans focusing on answering a specific clinical question could remarkably minimise and shorten the scan time. Recent fast techniques (compressed sensing) may also provide an alternative solution.   5. Body stature and body surface area of patients with MPS is usually small, although they are adult in age. The kilo-voltage and exposure protocols should be adjusted accordingly to minimise radiation.   6. Gadolinium might be contraindicated in some patients. Non contrast MRI protocols including native T1 parametric maps and non-contrast respiratory navigated 3D sequences may provide the necessary clinical information without need of gadolinium.   Limitation of radiological assessments in adult MPS   1. Learning difficulties 2. Communication barriers such a vision, hearing loss 3. Previous cardiac surgery can cause streak artifacts due to metal in sternum 4. MRI may be contraindicated in Cochlear implantation |

Abbreviations: MPS- mucopolysaccharidosis, CT- computed tomography, MRI- magnetic resonance imaging, MRA-magnetic resonance arteriography, CAD- coronary artery disease
