# Supplementary material for: Pre-operative Considerations in Adult Mucopolysaccharidosis Patients Planned for Cardiac Intervention
Source: Front Cardiovasc Med. 2022 Apr 4;9:851016. doi: 10.3389/fcvm.2022.851016 (PMC9013828; doi:10.3389/fcvm.2022.851016)
Supplement: Supplementary file 2 [file Table_2.docx]

Table Suppl 2: Pre-operative assessment questionnaire

| **Airway assessment** | - Bilateral patency of nasal cavities   - Assess ease of passing a fibre-optic scope for nasendoscopy and nasal intubation - Evidence of enlarged adenoids/tonsils - Evidence of acid reflux and need for a rapid sequence induction - Previous intubation and ventilation - Oral cavity   - Crowding of teeth   - Prominent teeth   - Mouth opening (Mallampati Grade) - Size of the tongue in relation to the oral cavity - Tongue mobility - Receding mandible - Mobility at cervical spine   - Flexion and extension   - X-ray flexion and extension views   - CT/MRI to rule out instability   - Spinal cord compression - Any abnormal deposits in the airway - Size and shape of the epiglottis / arytenoid cartilages / vocal cords, and the laryngeal inlet - Subglottic airway assessment   - By endoscopy under general anaesthetic   - By limited views from nasendoscopy   - Narrowing of or abnormalities in trachea-bronchial anatomy   - Length of the trachea from the vocal cords to the carinal bifurcation   - CT/MRI of the neck and thorax to assess anatomy and abnormalities of the trachea and bronchi, with review by a consultant radiologist   - 3D reconstruction of the airway   - 3D printing of the airway   - Trial intubation of 3D reconstruction     - For size of the bronchoscope     - For size and type of the endotracheal tube - Access to the front of the neck, in case of need for emergency tracheostomy   - Trachea centrally placed or deviated   - Ease of access to cricothyroid membrane   - Size of tracheostomy tube - Consent for:   - Failure to intubate   - Abandoning the procedure   - HDU or ICU admission for monitoring of airway and/or ventilation   - Controlled extubation |
| --- | --- |
| **Documentation of possible methods of and requirements for airway management / anaesthesia** | - Is IV access possible? - Is there a need for local anaesthetic cream or inhalational induction? - Awake/asleep endoscopy/intubation - Laryngeal mask airway   - Volume of upper airway GAG deposits - Endotracheal intubation   - Nasal/oral - Size of tubes   - Preparation of small tubes - Type of tube   - Microlaryngeal tube, microcuffed tube - Plan for secretion management - Choice of fibre-optic bronchoscopes - Rigid bronchoscope or Hopkins rod - Facilities to record pictures and live endoscopy videos for future reference - Choice of tracheostomy tubes - Plan for extubation - Role of IV steroids and endotracheal adrenaline prior to extubation - Extended recovery stay for monitoring - Need for:   - CPAP   - Transnasal humidified high-flow oxygen for apnoeic oxygenation - Postoperative destination   - General ward   - Monitored ward   - Critical care unit |

Table Suppl 3: Salford Metabolic Airway Score (70)

| **Serial number** | **Parameter** | **Measure** | **Score** | **Final Score** |
| --- | --- | --- | --- | --- |
|  | **MPS type** |  |  |  |
|  | **Mouth opening** | >5cm | 0 |  |
|  |  | 4-5cm | 1 |  |
|  |  | 3-4cm | 2 |  |
|  |  | <3cm | 3 |  |
|  |  |  |  |  |
| **2** | **Teeth protrusion on clinical exam and scans** | Non-protruding | 0 |  |
|  |  | Mild | 1 |  |
|  |  | Moderate | 2 |  |
|  |  | Severe | 3 |  |
|  |  |  |  |  |
| **3** | **Cervical spine mobility, stability** | unrestricted | 0 |  |
|  |  | 60-90 degrees flexion | 1 |  |
|  |  | 30-60 degrees flexion | 2 |  |
|  |  | < 30 degrees or unstable | 3 |  |
|  |  |  |  |  |
| **4** | **Tongue bulkiness on examination and Scan** | Normal | 0 |  |
|  |  | Mild | 1 |  |
|  |  | (Filling less than 1/3 of floor mouth) |  |  |
|  |  | Moderate | 2 |  |
|  |  | (Filling 1/3 to 1/2 of oral cavity) |  |  |
|  |  | Severe | 3 |  |
|  |  | (Filling more than 1/2 of oral cavity) |  |  |
|  |  |  |  |  |
| **5** | **Modified Mallampati grade [19]** | 1 | 0 |  |
|  |  | 2 | 1 |  |
|  |  | 3 | 2 |  |
|  |  | 4 | 3 |  |
|  |  |  |  |  |
| **6** | **Thyromental distance** | >6 cm | 0 |  |
|  |  | 5-6 cm | 1 |  |
|  |  | 4-5 cm | 2 |  |
|  |  | <4 cm | 3 |  |
|  |  |  |  |  |
| **7** | **Larynx height epiglottis to soft palate** | >4cm | 0 |  |
|  |  | 3-4cm | 1 |  |
|  |  | 2-3cm | 2 |  |
|  |  | <2cm | 3 |  |
|  |  |  |  |  |
| **8** | **Epiglottis bulkiness** | Normal | 0 |  |
|  |  | (Filling less than 1/3 of oropharynx) |  |  |
|  |  | Mild | 1 |  |
|  |  | (Filling 1/3 to 1/2 of oropharynx) |  |  |
|  |  | Moderate | 2 |  |
|  |  | (Filling 1/2 to complete oropharynx) |  |  |
|  |  | Severe | 3 |  |
|  |  | (Filling entire oropharynx) |  |  |
|  |  |  |  |  |
| **9** | **Supraglottis bulkines** | Normal | 0 |  |
|  |  | (Filling less than 1/3 of laryngopharynx) |  |  |
|  |  | Mild | 1 |  |
|  |  | (Filling 1/3 to ½ of laryngopharynx) |  |  |
|  |  | Moderate | 2 |  |
|  |  | (Filling ½ to complete laryngopharynx) |  |  |
|  |  | Severe | 3 |  |
|  |  | (Filling entire oropharynx) |  |  |
|  |  |  |  |  |
| **10** | **Glottis bulkiness** | Normal | 0 |  |
|  |  | (Filling less than 1/3 of glottis) |  |  |
|  |  | Mild | 1 |  |
|  |  | (Filling 1/3 to 1/2 of glottis) |  |  |
|  |  | Moderate | 2 |  |
|  |  | (Filling 1/2 to complete glottis) |  |  |
|  |  | Severe | 3 |  |
|  |  | (Filling entire glottis) |  |  |
|  |  |  |  |  |
| **11** | **Sub glottis diameter at cricoid level** | >7mm | 0 |  |
|  |  | 6-7mm | 1 |  |
|  |  | 5-6mm | 2 |  |
|  |  | <5mm | 3 |  |
|  |  |  |  |  |
| **12** | **Tracheo – malacia or tracheal stenosis** | No narrowing | 0 |  |
|  | **(degree of narrowing)** |  |  |  |
|  |  | 50-75% lumen narrowing | 1 |  |
|  |  | 75-99% lumen narrowing | 2 |  |
|  |  | 100% lumen narrowing | 3 |  |
|  |  |  |  |  |
| **13** | **Tracheal tortuosity** | None | 0 |  |
|  |  |  |  |  |
|  |  |  |  |  |
|  |  | present | 3 |  |
|  |  |  |  |  |
| **14** | **FEV1%** | >80% | 0 |  |
|  |  | 60-79% | 1 |  |
|  |  | 40-59% | 2 |  |
|  |  | <40% | 3 |  |
|  |  |  |  |  |
| **15** | **FVC%** | >80% | 0 |  |
|  |  | 60-79% | 1 |  |
|  |  | 40-59% | 2 |  |
|  |  | <40% | 3 |  |
