# Supplementary material for: Pre-operative Considerations in Adult Mucopolysaccharidosis Patients Planned for Cardiac Intervention
Source: Front Cardiovasc Med. 2022 Apr 4;9:851016. doi: 10.3389/fcvm.2022.851016 (PMC9013828; doi:10.3389/fcvm.2022.851016)

Figure Suppl 1-4: Airways in adult MPS patients.

Figure Suppl 1: Nasendoscopy in an MPS II showing high larynx, bulky supraglottis


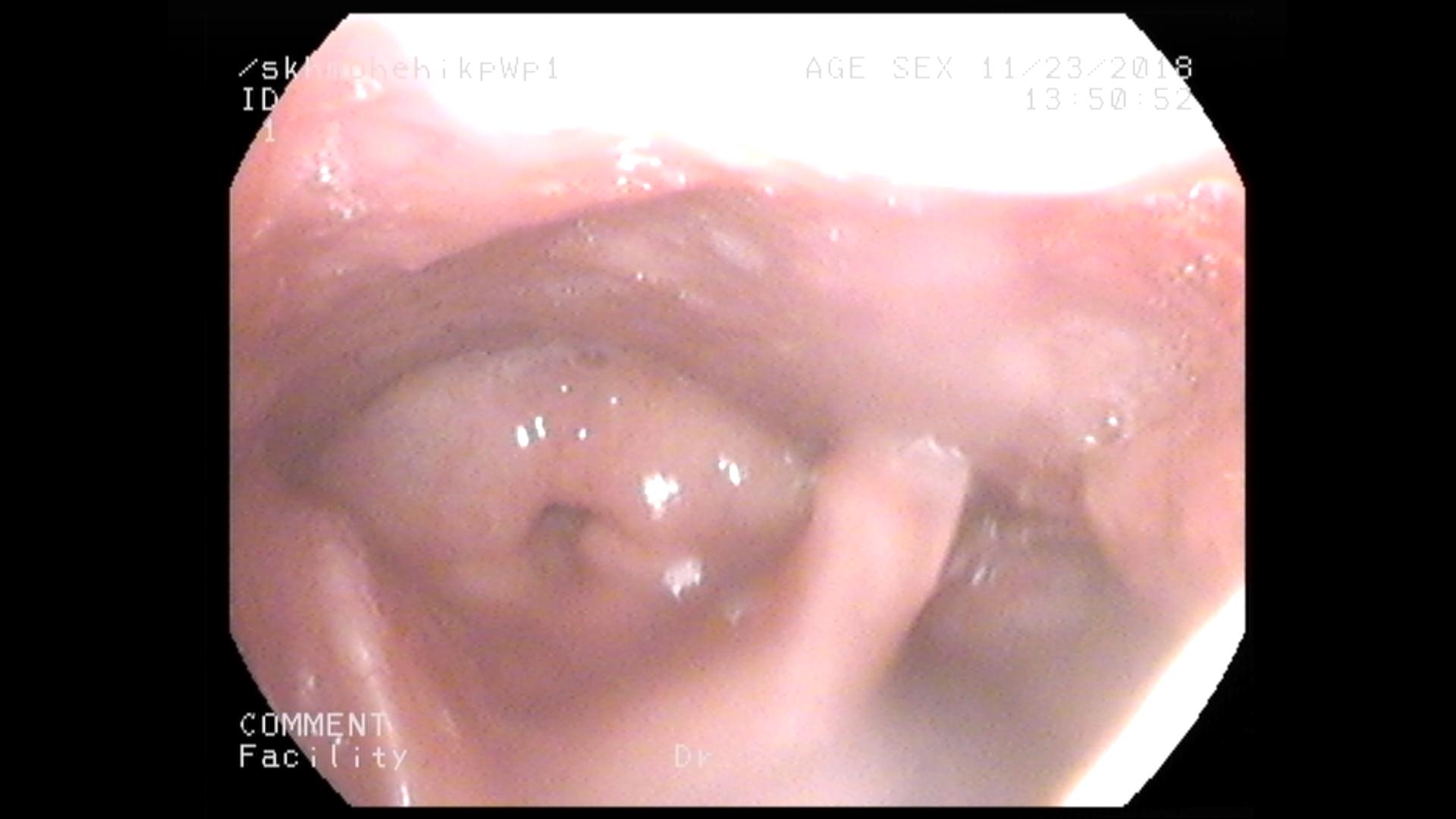

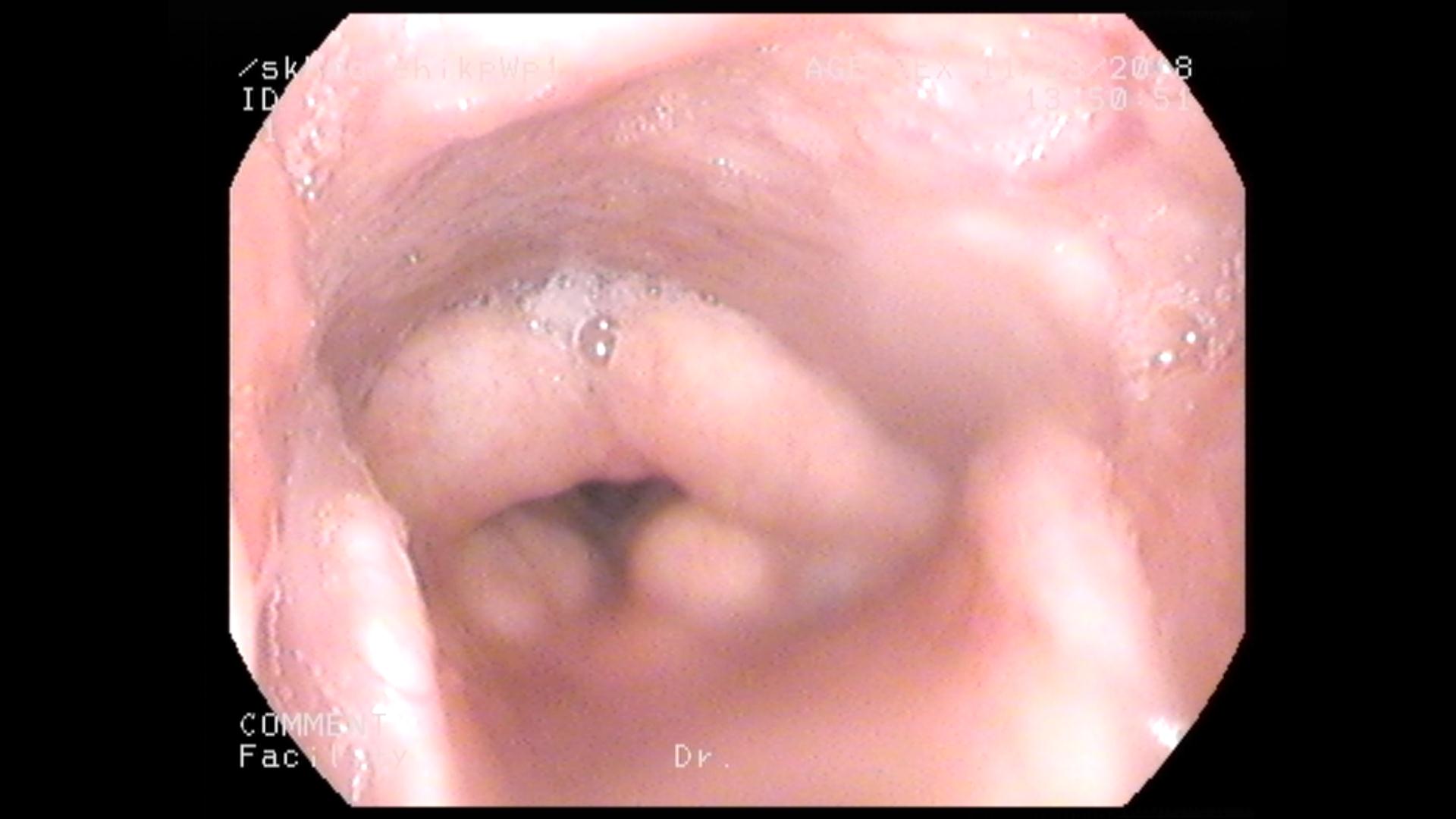


Figure Suppl 2: 3-dimensional reconstruction of the airway in MPS I showing mildly curved trachea


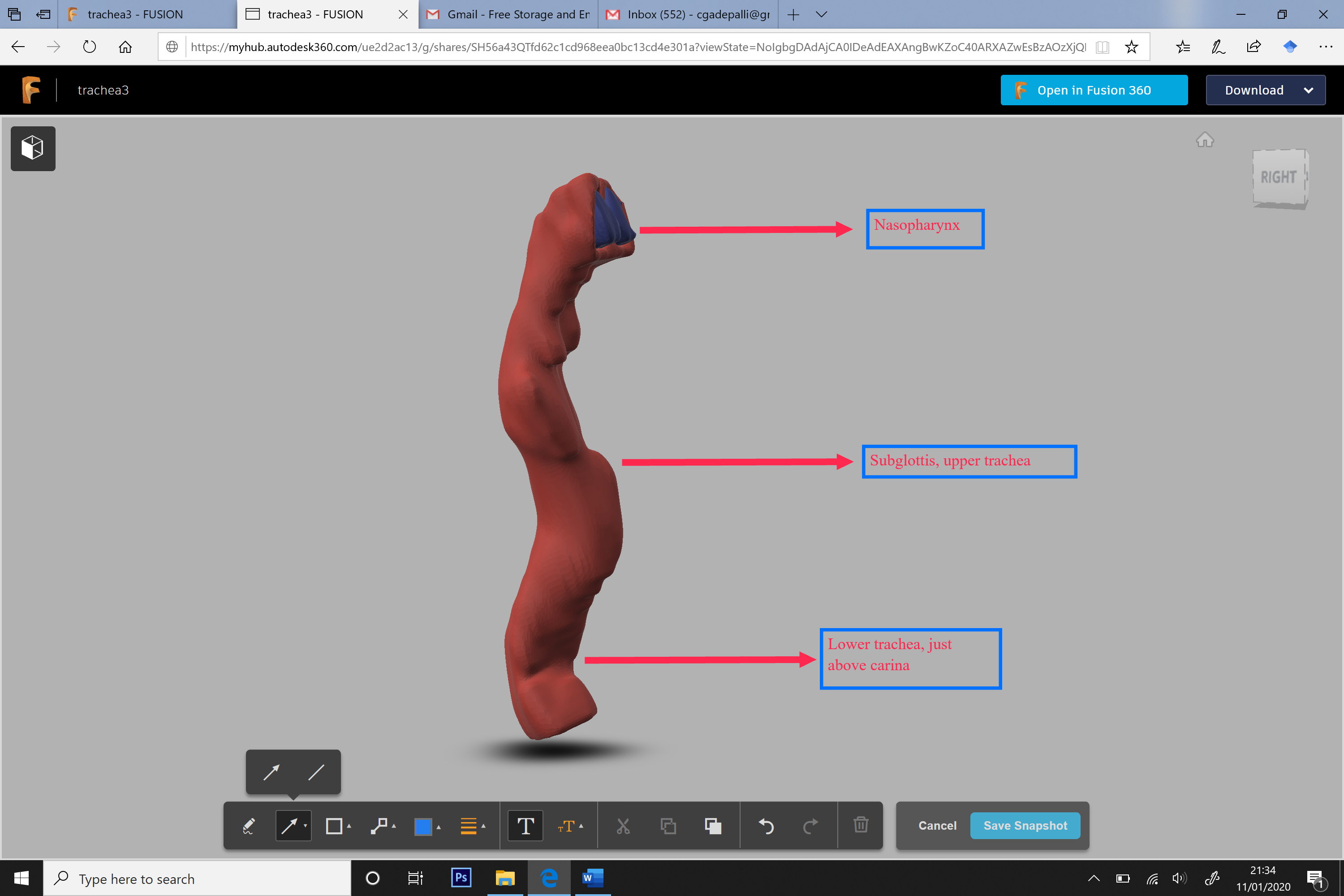


Figure Suppl 3: 3-dimensional reconstruction of airway in MPS VI, showing tortuous trachea and malacia in anterio-posterior and lateral view


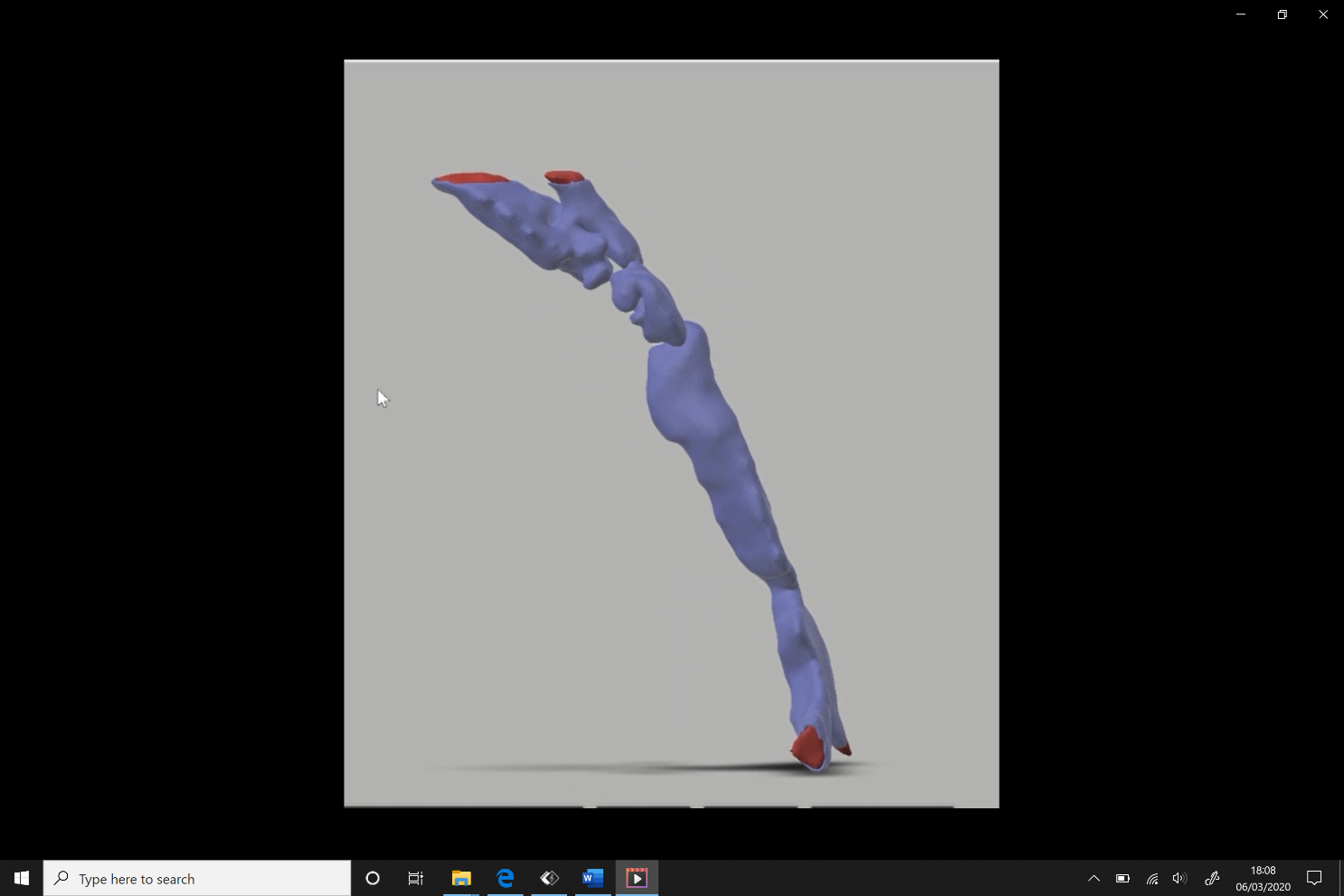

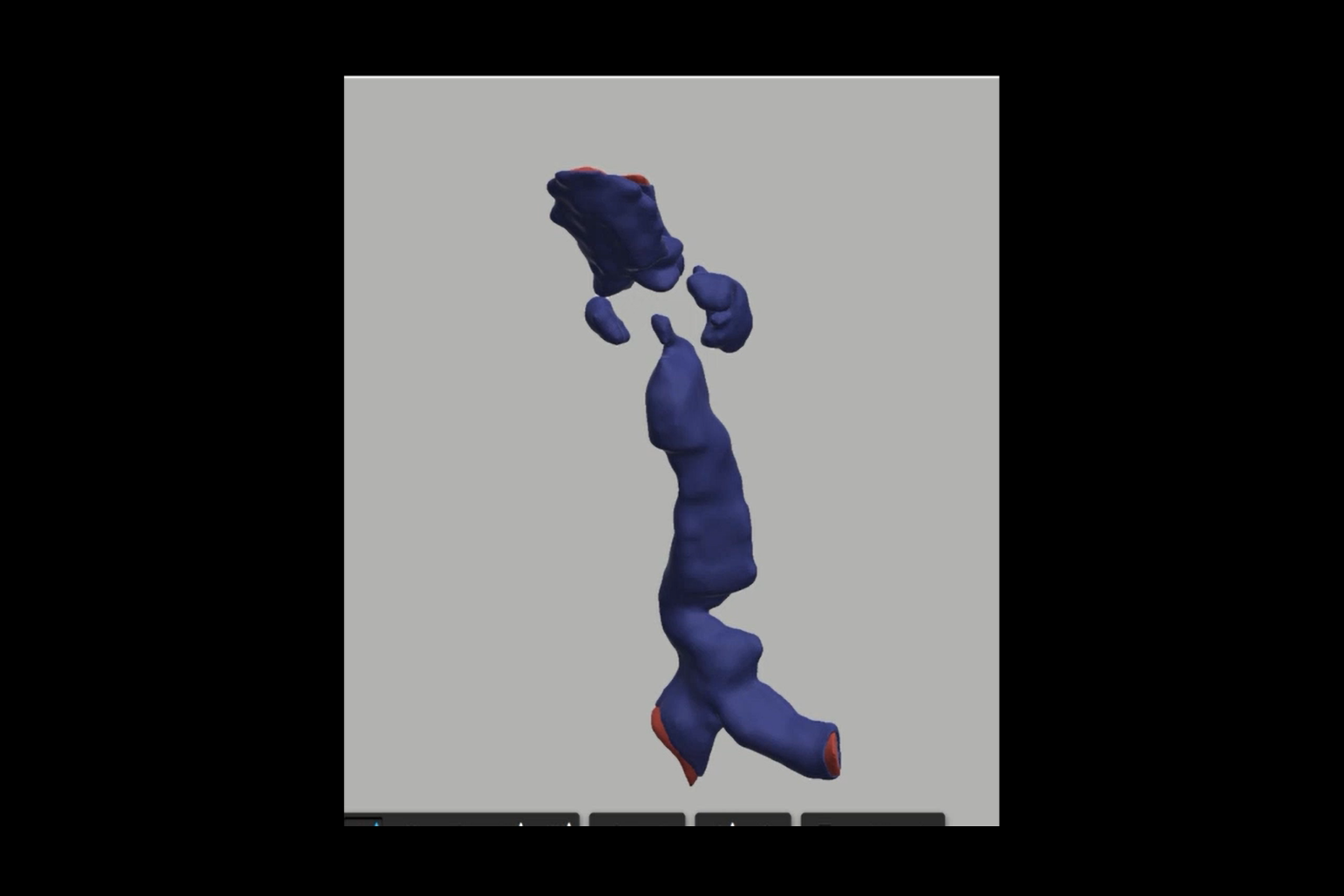


Figure Suppl 4: 3-Dimensional reconstruction of airway in Mucopolysaccharidosis II in antero-posterior, lateral view showing narrowing of trachea and flattened trachea suggestive of tracheomalacia


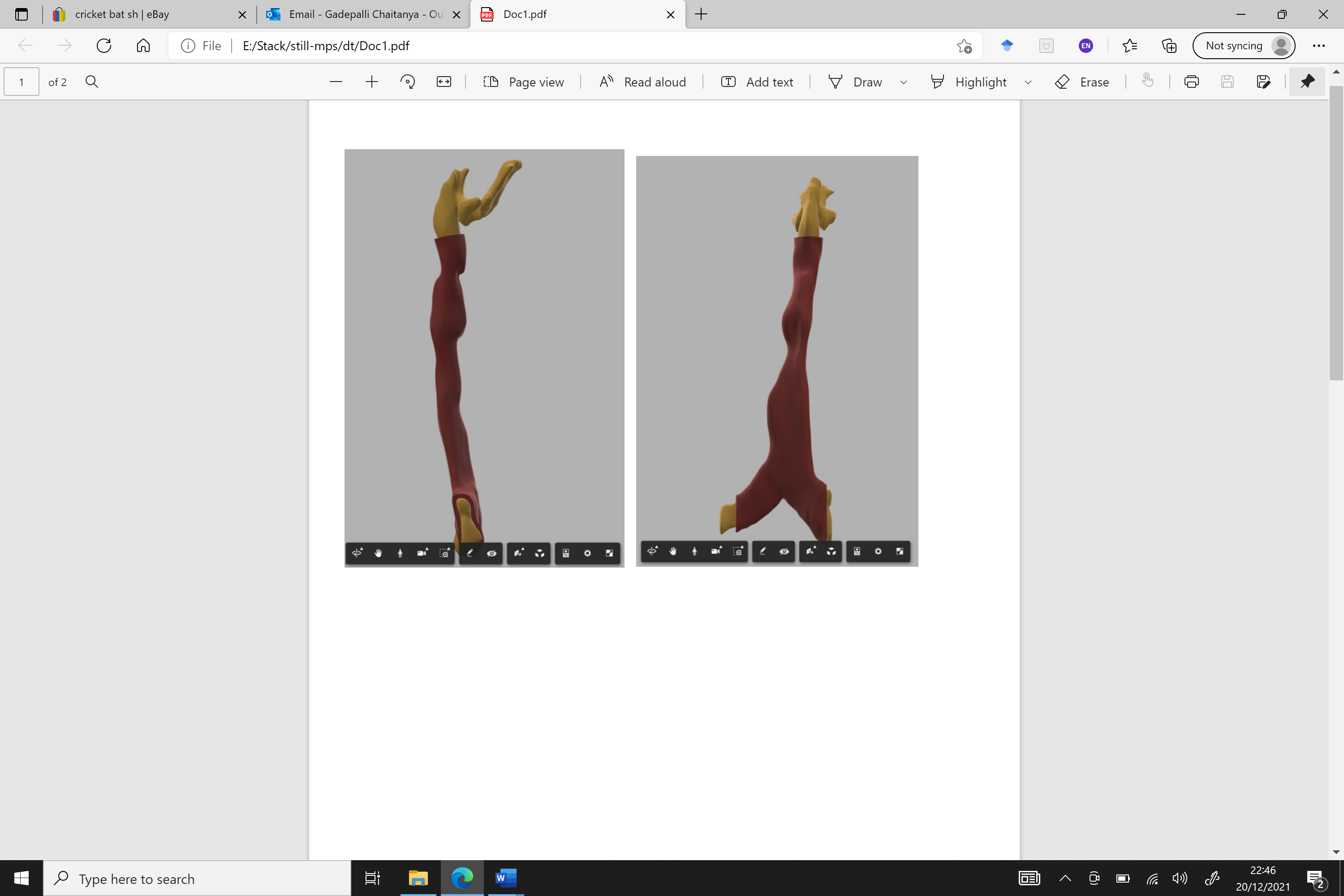

Supplement: Supplementary file 3 [file Data_Sheet_1.docx]
